# Long-term follow-up in patients with HIV vaccinated with pandemic influenza A(H1N1)/09 AS03-adjuvanted split virion vaccine and seasonal trivalent influenza split virion vaccine

**DOI:** 10.3402/iee.v3i0.20766

**Published:** 2013-08-30

**Authors:** Karlis Pauksens

**Affiliations:** Section of Infectious Diseases, Department of Medical Sciences, Uppsala University, Uppsala, Sweden

**Keywords:** HIV, influenza vaccine, pandemic, long-term immunity, TIV

## Abstract

**Introduction:**

In Sweden in 2009, two doses of the pandemic influenza A(H1N1)/09 AS03-adjuvanted split virion vaccine were recommended for those with HIV infection along with one dose of seasonal trivalent influenza vaccine (TIV). At that time, no data for HIV patients and their response to the adjuvanted vaccine were available.

**Methods:**

Forty-two HIV-infected individuals were vaccinated with the pandemic vaccine on study days 0 and 28. Twenty-one of them received TIV on day 56 and 21 did not. Serum samples were taken at these time points, and also on day 86 and after 1 year for serologic analyses.

**Results:**

Before vaccination, none of the 42 patients had putatively protective levels of antibodies (haemagglutination inhibition [HI] titres ≥1:40) to the pandemic-like strain A/California/7/2009 H1N1. After dose 1, the seroprotection rate (SPR) and seroconversion rate (SCR) were both 69% (29 of 42). After dose 2, the SPR and SCR were 89 and 86%, respectively. At 1 year, 10 (34%) of 29 had protective antibodies and 16 (62%) of 26 who had had protective antibody levels had lost them. There was a retained factor increase of the geometric mean titre (GMT) of 3.9.

Serological analyses could be performed in 19 subjects who were vaccinated with TIV and in 21 who were not. Protective antibodies to the three strains before vaccination were 20–37%. The SCR was 26% to A/Brisbane/59/2007 H1N1, 47% to A/Uruguay/10/2007/ H3N2 and 42% to B/Brisbane/60/2008. At 1 year, the factor increase of GMT was 1.8 to the two influenza A strains.

**Conclusion:**

Two doses of adjuvanted influenza vaccine improved the SCR and the SPR among HIV-infected subjects. Long-term follow-up indicates revaccination in the next influenza season whether they received an adjuvanted or non-adjuvanted influenza vaccine.

Influenza epidemics and pandemics result in increased morbidity and mortality in the population and also among those with impaired immune defence, including HIV-infected individuals (1, 2). Many guidelines recommend annual influenza vaccination to these groups, although the response to the vaccine is impaired compared with in healthy individuals (3, 4). When the outbreak of the pandemic influenza in 2009 occurred, the new influenza A(H1N1)/09 AS03-adjuvanted split virion vaccine Pandemrix™ was recommended to all individuals in Sweden, and for those with impaired immune defence two doses were recommended (5, 6). At that time, there were no data available concerning the immunogenicity of the adjuvanted pandemic vaccine among HIV-infected individuals, or concerning the duration of the antibody response. The seasonal non-adjuvanted trivalent influenza vaccine (TIV) was also recommended for the same season, but it was unclear if the vaccine should be given concomitantly with the pandemic vaccine or separately. It was also unclear if the seasonal influenza vaccine could boost the response to the adjuvanted pandemic vaccine or, vice versa, if the adjuvanted vaccine affects the response to the TIV.

Therefore, we vaccinated with two doses of the pandemic vaccine 4 weeks apart, and after a further 4 weeks the TIV was given. We measured the haemagglutination inhibition (HI) antibody titres before and 4 weeks after each vaccination and at long-term follow-up after 1 year to both the pandemic strain and the strains in the TIV.

## Materials and methods

All patients with HIV infection followed at the Department of Infectious Diseases at Uppsala University Hospital, Sweden, were offered participation in the study. All patients were adults older than 18 years of age. No exclusion criteria were used except that patients who knew that they could not come for follow-up after vaccination were not included. The study was approved by the Regional Ethics Committee and undertaken in compliance with Good Clinical Practice Guidelines and The Declaration of Helsinki. Written informed consent was obtained from all participants.

In Sweden, the influenza A(H1N1)/09 AS03-adjuvanted split virion vaccine Pandemrix™ from GlaxoSmithKline was recommended to all citizens free of charge (6). For individuals with increased risk for severe influenza infection, including HIV-infected individuals, two doses were recommended with at least 3 weeks between doses (4).

The split vaccine contained 3.75 µg of haemagglutinin antigen from the inactivated influenza A/California/7/2009 (H1N1)-derived strain and the Adjuvant System 03 (AS03) composed of squalene (10.69 mg), DL-α-tocopherol (11.86 mg), and polysorbate 80 (4.86 mg), and it was given intramuscularly in the deltoid muscle in the non-dominant arm.

Annual vaccination with the seasonal influenza vaccine is also recommended in immunocompromised patients in Sweden. The seasonal influenza vaccines used for the 2009–2010 transmission season were non-adjuvanted and contained 15 µg of haemagglutinin from each of the strains A/Brisbane/59/2007 H1N1-like virus, A/Brisbane/10/2007 (H3N2)-like virus (A/Brisbane/10/2007 or A/Uruguay/716/2007 vaccine virus), and B/Brisbane/60/2008-like virus.

### Vaccination and serology

At baseline (day 0), the first dose of the adjuvanted A(H1N1) vaccine was given, and the second dose was given on day 28. Vaccination with the pandemic strain began on 21 October 2009. The seasonal influenza vaccine was given on day 56 and started in mid-December 2009. Serum for antibody analyses was collected at days 0, 28, and 56 and at 1 year. For those who received a seasonal influenza vaccination, serum was also collected on day 86. All available blood samples were analysed for the pandemic strain and for the three influenza strains in the seasonal influenza vaccine at each time point.

The assays for measurement of antibody response were performed by GSK Biologicals. All serum samples were collected and frozen at the hospital. When the last serum sample was taken, they were blinded, coded, and sent to the laboratory concomitantly for analysis. The antibody titres against the strains in the pandemic and seasonal influenza vaccines were measured by a validated HI assay as described by the World Health Organization (WHO) Collaborating Center for Influenza and the US Centers for Disease Control and Prevention (7). Briefly, the serum samples were treated by enzymatic treatment and heated to destroy non-specific inhibitors. Haemagglutination was performed in a microtitre test using chicken erythrocytes with the A/California/7/2009 (H1N1) strain as antigen for the pandemic vaccine and A/Brisbane/59/2007H1N1 and A/Uruguay/716/2007H2N3 strains as antigens for the seasonal influenza vaccine. HI assays were performed in duplicates for each sample using serial twofold dilutions with a starting dilution of the treated serum of 1:10. The sample titre was the highest dilution that completely inhibited haemagglutination. Titres below the detection limit of 1:10 were assigned a value of 1:5 in the calculations. An HI reciprocal titre ≥40 was regarded as protective against influenza infection for 50% of the vaccines and defined as seroprotection (8). The response to vaccination (seroconversion) was defined as at least a fourfold increase in antibody titre after vaccination or, if the pre-vaccination HI reciprocal titre was <10, a post-vaccination titre of HI ≥ 40.

In the HI assay used for the influenza B/Brisbane/60/2008-like virus, the B viruses were split with ether treatment (the A strains were not). Due to this treatment, Type B virus titres are typically higher than Type A virus titres and are not comparable. Therefore, only the SCRs of the influenza B strain are shown.

### Statistical analysis

The antibody titres were expressed as geometric mean titres (GMT) before and after vaccination. In comparisons between or within different patient groups, the non-parametric Mann–Whitney *U* test, χ^2^ test, Wilcoxon's signed rank test, paired sign test, Fisher's exact test, and simple regression test were used when appropriate.

## Results

### Pandemic vaccine

Forty-four HIV-infected patients were included in the study and vaccinated with the influenza A(H1N1)/09 AS03-adjuvanted split virion vaccine, and 42 of them were eligible for serologic analyses. Patient characteristics are shown (Table 1) for those 42 patients who were included in the serological analyses. The mean age was 47 ± 13.3 years, and the median age was 46 years, with a range of 25–82 years. No patient had received chemotherapy within the last 3 years, and only one patient with renal impairment had a low dose of cyclosporine and prednisone (10 mg).

**Table 1:** Basic characteristics of HIV-infected patients

At vaccination	All (*n*=42)	18–44 years (*N*=19)	45–59 years (*N*=16)	60–82 years (*N*=7)
Years, mean± (SD)	47±13.3	35±4.7	52±4.0	69±8.5
Male/female	22/20	3/16	13/3	6/1
HAART,* yes (%)	36 (86%)	15 (79%)	14 (88%)	7 (100%)
CD4+ (cells/mm^3^)	473±193	416±199	528±196	484±162
HAART, no (%)	6 (14%)	4 (12%)	2 (21%)	0
CD4+ (cells/mm^3^)	555±105	549±133	567±37	–
Hepatitis B (all)	15 (36%)	10 (53%)	3 (19%)	2 (29%)
Chronic or active	2 (5%)	2 (11%)	0	0
Hepatitis C	5 (12%)	1 (5%)	4 (25%)	0

* Highly active antiretroviral therapy.

At baseline before vaccination at day 0, none had protective antibody levels to A/California/7/2009 H1N1, and low levels of antibodies were found in 5 (12%) of 42 (HI titres between 10 and 20). At day 28 before dose 2, 29 (69%) of 42 had protective HI titres ≥40 and a seroconversion response to the vaccine (Table 2).

**Table 2:** Haemagglutination inhibition titres after vaccination with H1N1 A/California 2009-like strain

Vaccination	All	18–44 years	45–59 years	≥60 years
Day 0, no.^a^	42	19	16	7
GMT^b^	5.8	5.6	6.3	5.0
Range	5–20	5–20	5–20	0
HI^c^ titre ≥40	0	0	0	0
Day 28, no.	42	19	16	7
GMT	56.1	77.1	61.7	19.1
Range	5–640	5–453	5–640	5–80
HI titre ≥40 no.	29 (69%)	15 (79%)	10 (63%)	4 (57%)
Factor increase of GMT	9.8	13.8	9.7	3.8
Seroconversion rate	29 (69%)	15 (79%)	10 (63%)	4 (57%)
Day 56, no.	37	15	15	7
GMT	105.0	145.9	139.4	28.2
Range	5–1280	20–640	40–1280	5–80
HI titre ≥40 no.	33 (89%)	14 (93%)	15 (100%)	4 (57%)
Factor increase of GMT	17.9	25.4	21.6	5.6
Seroconversion rate	32 (86%)	14 (93%)	14 (93%)	4 (57%)
1 year, no.	29	12	11	6
GMT	23.3	32.6	21.3	14.1
Range	5–320	5–320	5–80	5–80
HI titre ≥40 no.	10 (34%)	5 (42%)	4 (36%)	1 (17%)
Factor increase of GMT	3.9	5.8	3.0	2.8

a no.=number

b GMT = geometric mean titre

c HI = haemagglutinin inhibition assay.

Twenty-eight days (day 56) after the second dose, 33 (89%) of 37 had HI titres ≥40, and 32 (86%) had a seroconversion response. Of the five patients who could not be tested after the second dose, three had HI titres ≥40 and a seroconversion response, whereas two had not after the first dose of vaccine on day 28.

After the first dose of vaccine, the GMT increased from 5.8 to 56.7 with a factor increase of 9.8; and after the second dose, the GMT was 105.0 with a factor increase of 17.9. The greatest increase of GMT was seen in the younger patients (Table 2).

At 1 year from baseline, 10 (34%) of 29 who were eligible for serologic analyses had HI titres ≥ 40, and 19 (66%) had not. In total, 16 (62%) of 26 who had had HI titres ≥40 at day 56 and who came at 1-year control had lost their protective antibody levels, whereas 3 of the 29 patients never had had protective antibody levels. When compared with day 0, there was a retained GMT of 23.3 and a factor increase of 3.9 (Table 2).

Different HIV-related factors were studied if they correlated to vaccine response. Only higher age was found to be significantly correlated with reduced response to vaccination *p*<0.001 (simple regression analysis). This is also shown for GMT levels in Table 2. There was no significant correlation if patients were on antiretroviral medication or not, duration of HIV infection, CD4 count at nadir or at vaccination or previous HIV copies before initiating antiretroviral therapy.

Whether vaccination with seasonal influenza vaccine gave a booster response to the pandemic strain was also studied. In 19 patients who were vaccinated with seasonal influenza vaccine on day 56, HI titres to A/California/7/2009 H1N1 were analysed 1 month later on day 86 (Table 3). During this month, the GMT declined from 82.7 to 45.3, *p*<0.001 (Wilcoxon's signed rank test), and four (25%) of 16 lost their protective antibody titres.

**Table 3:** Haemagglutination inhibition titres against A/California in HIV patients before and after vaccination with seasonal influenza vaccine

A/California	All	18–44 years	45–59 years	≥60 years
No.^a^	19	7	8	4
Age±SD (years)	48±13.1	37±2.3	50±3.8	70±8.7
Day 56^b^, GMT^c^	77.2	107.8	91.3	30.8
Range	14–640	20–640	40–160	14–80
HI titre ≥40 no.	16 (84%)	6 (86%)	8 (100%)	2 (50%)
Factor increase of GMT^d^	13.1	20.7	13.4	6.2
Day 86, GMT	45.3	48.7	61.5	21.8
Range	10–226	10–226	28–160	10–40
HI titre ≥40 no.	12 (63%)	4 (57%)	6 (75%)	2 (50%)
Factor increase of GMT^d^	7.7	9.4	9.0	4.4

a no.=number

b corresponds from baseline day 0

c GMT = geometric mean titre

d compared with day 0.

### Seasonal influenza vaccination

Seasonal influenza vaccine was given to 22 patients on day 56. One patient could not be followed because of travel abroad and in two patients antibody titres from day 86 could not be assessed despite repeated analyses because of interfering factors in the serum. Antibodies to the influenza strains in the seasonal influenza vaccine were measured for all patients at days 0, 28 (not shown), 56 and 86 and at 1 year (Table 4).

**Table 4:** Haemagglutin inhibition titres to the seasonal influenza A strains A/Brisbane and A/Uruguay in HIV patients vaccinated and not vaccinated with seasonal influenza vaccine

	A/Brisbane		A/Uruguay	
		
	Vaccinated	Not vaccinated	Vaccinated	Not vaccinated
Day 0, no.^a^	19	21	19	21
Age±SD years	48±13.1	46 + 13.8	48±13.1	46 + 13.8
GMT^b^	10.6	11.0	14.1	12.6
Range	5–113	5–80	5–80	5–80
HI^c^ titre ≥40, no.	4 (21%)	4 (19%)	7 (37%)	5 (24%)
Day 56, GMT	12.4	13.5 no. 16	12.9	12.4 no. 16
Range	5–160	5–80	5–80	5–80
HI titre ≥40 no.	4 (21%)	4 (25%)	7 (37%)	4 (25%)
Day 86, GMT	29.3		56.5	
Range	5–160		5–320	
HI titre ≥40 no.	10 (53%)		12 (63%)	
Factor increase of GMT	2.8		4.4	
Seroconversion rate	5 (26%)		9 (47%)	
0–1 year follow-up no.	14	15	14	15
Day 0, GMT	13.1	10.0	19.0	11.5
HI titre ≥40 no.	5 (36%)	3 (20%)	7 (50%)	3 (20%)
Day 86^d^, GMT	31.5		75.8	
HI titre ≥40 no.	7 (54%)		10 (77%)	
Seroconversion rate	4 (31%)		7 (54%)	
1 year, GMT	23.2	9.1	34.5	8.3
HI titre ≥40 no.	6 (43%)	2 (13%)	8 (57%)	2 (13%)
Factor increase of GMT	1.8	0.9	1.8	0.7

a no.= number

b GMT= geometric mean titre

c HI= haemagglutinin inhibition assay

d corresponds to after seasonal influenza vaccination.

At baseline, protective antibody levels (HI titres ≥40) to A/Brisbane/59/2007H1N1-like virus and A/Uruguay/10/2007/H3N2-like virus were 10 (24%) and 13 (31%) of 42 subjects, respectively. No significant increase in antibody titres was seen during vaccination with the pandemic vaccine from day 0 to 56 for the two influenza A strains (Table 4).

At day 56 and before vaccination with TIV, four (21%) of 19 subjects had HI titres ≥40 to A/Brisbane/59/2007 H1N1-like virus, and seven (37%) to A/Uruguay/10/2007/ H3N2-like virus. At day 86, 1 month after vaccination, 10 (53%) of 19 had HI titres ≥40 to A/Brisbane/59/2007 H1N1-like virus, and 10 (63%) to A/Uruguay/10/2007/ H3N2-like virus. A vaccine response with at least a fourfold titre increase and HI titres ≥40 was found in 5 (26%) of 19 and 9 (47%), respectively. The corresponding SCR for B/Brisbane/60/2008-like virus was 8 (42%) of 19.

At 1 year, 14 of 19 vaccinated subjects were retested, 6 (43%) had HI titres ≥40 to A/Brisbane/59/2007 H1N1-like virus and 8 (57%) to A/Uruguay/10/2007/ H3N2-like virus. The factor increase of GMT at 1 year compared with day 0 was 1.9 and 2.0, respectively. Fifteen non-vaccinated subjects were also retested at 1 year, and had a corresponding factor increase of GMT 0.7 and 0.9 of the influenza A strains.

Whether pre-existing protective antibodies to the two influenza A strains in the seasonal influenza vaccine at baseline had a significant impact on the antibody response to the pandemic vaccination was also studied. Only a weak correlation was found for those 12 subjects who had protective antibodies to A/Uruguay/10/2007/ H3N2-like virus at day 56, who also had a significantly greater GMT for the A/California/7/2009 H1N1 strain (190.2 vs. 78.9 (*p*<0.044), Mann–Whitney *U* test) than the 25 who had no protective antibody titres.

## Discussion

In this study, nearly 70% of HIV-infected individuals receiving the ASO3-adjuvanted split virion vaccine with 3.75 µg amount of H1N1 haemagglutinin antigen achieved a protective antibody response after one dose of vaccine. The response after two doses showed a further increase in the number of individuals who reached a protective antibody level (HI titre ≥ 40) of almost 90%. The best antibody responses were seen in younger adults, whereas a lower antibody response measured as GMT and a lower seroprotection rate (SPR) was seen with higher age. Although only a small number of patients older than 60 years were studied, no further increase in the SPR was seen after the second dose of vaccine, and only a modest increase in GMT.

The protection rate was comparable with those of other studies with unselected HIV-infected individuals who received adjuvanted vaccines and had an impaired response to vaccination with greater age but a somewhat lower response than in clinical trials, where a selected population of HIV-infected patients were included (9–11).

In comparison with studies in healthy individuals, where a single dose of the adjuvanted vaccine showed protection rates in up to 98% of the vaccinated adults, the response rate among HIV-infected individuals seems to be lower (12). However, in studies where the population was split up into different age groups, the response to vaccination also declined with greater age, and among those older than 65 years of age neither the SPR nor SCR reached more than 81% (13).

Long-term follow-up at 1 year after the first dose of adjuvanted vaccine showed that there was a retained factor increase of GMT with 3.9 but that 62% of the patients had lost their protective antibody levels. Although we did not include healthy controls, a recent study found that HIV-infected individuals had an impaired antibody response measured as GMT and also lost protective antibody titres more often than the healthy individuals when followed for 6 months (14). The impaired response was attributed to defects in B-cell function (15). This indicates that the majority of HIV-infected patients should be revaccinated after 1 year to improve protection against the pandemic influenza strain. However, there is also a recent clinical trial with selected HIV-infected individuals who had retained antibody levels at similar levels as healthy controls (16).

Whether vaccination with the AS03-adjuvanted pandemic vaccine had any impact on the antibody levels to the influenza A strains included in the seasonal influenza vaccine was studied. No significant changes in the HI titres or SPRs for the A/Brisbane/59/2007H1N1-like virus and A/Uruguay/10/2007/H3N2-like virus were found until the seasonal influenza vaccine was given on day 56. In contrast, for the influenza B/Brisbane/60/2008-like virus, there was a significant increase in HI titres from baseline until day 56 with a factor increase of GMT with 1.8 (data not shown). The difference could be explained by the HI assay methods difference, where influenza A antigens are native antigens, whereas the B strain virus is split and ether treated and seems to be less specific and thus give higher HI titres.

We also studied if vaccination with the seasonal influenza vaccine had any booster effect of the HI titres for the A/California/7/2009 H1N1 strain from day 56 until day 86, but no such effects could be found. On the contrary, there was a decline of antibody titres for the pandemic strain, and 25% of the patients lost their protective antibody titres a month after vaccination with the seasonal influenza vaccine.

In another study, previous vaccination with seasonal influenza vaccine showed increased numbers of HIV-infected individuals who had protective antibody titres against the pandemic influenza strain before vaccination (11, 17). This was most pronounced if TIV was given a month before vaccination with the pandemic vaccine but was also found if the vaccination was performed several years ago. However, in this study we could not verify this finding since none had pre-existing protective antibodies to the A/California strain before vaccination, although 20–35% of the patients had protective antibodies to the influenza strains included in the seasonal influenza vaccine. The differences in the results could perhaps be explained by differences in the laboratory methods used and the specificity of the antigens used in the tests.

The SCR and the increase of GMTs after vaccination with seasonal influenza vaccine were impaired compared with the response to the adjuvanted pandemic vaccine and did not reach over 50% for any of the three strains, and it was as low as 26% for the influenza A/Brisbane//59/2007 (H1N1) strain and lower than with the adjuvanted vaccine that also showed a further increased response rate after the second dose. This has not been found to be the case after repeated vaccination with the non-adjuvanted influenza vaccine in either healthy individual or immunocompromised patients (18, 19). A repeated dose had no or a very limited effect on increasing the response rate among healthy individuals and in immunocompromised individuals (19). Because of the low number of included individuals, a differentiation into age groups was not fruitful for the seasonal influenza vaccine.

In comparison with the pandemic strain, a great proportion of patients had protective pre-existing antibodies to the strains included in the seasonal influenza vaccine (20–37%), probably caused by previous vaccination and/or natural influenza infection. At follow-up, at 1 year predominantly, these patients with pre-existing antibodies at day 0 retained their antibodies more frequently whether they had been vaccinated or not, whereas those without pre-existing antibodies and who were vaccinated with TIV lost more frequently their putatively protective levels of antibodies. However, because of the small number of patients, statistical calculations were not fruitful, and this could only be reported as an observation.

At follow-up at 1 year, there was a small retained increase in antibody levels measured as GMT and in seroprotection among those vaccinated with seasonal influenza compared with those who were not, indicating some beneficial effect of annual influenza vaccination on HIV-infected individuals.

In this study, self-reporting of adverse event and influenza-like illness was asked at the visits for blood sampling. No patient reported any influenza-like illness during the study period. The pandemic influenza period declined very sharp at the end of the year 2009/beginning of 2010 and different epidemiological surveillance systems showed that there were no other seasonal influenza epidemics that appeared during this season. These data indicate that the obtained antibody responses were mainly from vaccination and the likelihood for natural infection was small during this time period.

The adverse events were collected by self-reporting, and no severe adverse event was reported except for local reactions.

The used AS03-adjuvanted split virion vaccine with 3.75 µg amount of H1N1 haemagglutinin antigen seems to give the best antibody response of used pandemic vaccines (20). However, an association with increased rates of narcolepsy in children and young individuals following vaccination has been reported. While investigations into this association continue, European Regulatory Authorities have introduced restrictions for the use of this vaccine in persons younger than 20 years of age (21).

The limitations of the study are the rather small number of patients included and that not all could be followed until the next season. However, the number included corresponds to a great proportion of available patients. The strength of the study was that we could compare the immunogenicity of an adjuvanted vaccine with a non-adjuvanted influenza vaccine in the same individual. We could also follow up the antibody titres during 1 year and who received TIV and not.

## Conclusion

After two doses of adjuvanted pandemic vaccine, almost 90% of the HIV-infected individuals achieved protective antibody levels against the pandemic strain, with the best response in younger individuals. The response to the non-adjuvanted seasonal influenza vaccine was lower, with less than 50% SCRs to the included influenza strains. Long-term follow-up showed that most patients lost their seroprotective antibody levels after both the pandemic vaccine and the seasonal influenza vaccine, indicating that revaccination is necessary for the next influenza season.
